# The Role of Rosmarinic Acid in Cancer Prevention and Therapy: Mechanisms of Antioxidant and Anticancer Activity

**DOI:** 10.3390/antiox13111313

**Published:** 2024-10-28

**Authors:** Adam Kowalczyk, Carlo Ignazio Giovanni Tuberoso, Igor Jerković

**Affiliations:** 1 Department of Pharmacognosy and Herbal Medicines, Faculty of Pharmacy, Wroclaw Medical University, 50-556 Wroclaw, Poland; 2 Unit of Pharmaceutical, Pharmacological and Nutraceutical Sciences, Department of Life and Environmental Sciences, University of Cagliari, 09124 Cagliari, Italy; tuberoso@unica.it; 3 Department of Organic Chemistry, Faculty of Chemistry and Technology, University of Split, 21000 Split, Croatia; igor@ktf-split.hr

**Keywords:** rosmarinic acid, antioxidant activity, anticancer activity, cancer prevention

## Abstract

Rosmarinic acid (RA), a polyphenolic compound found in herbs, such as rosemary, basil, and mint, has garnered significant attention due to its potent antioxidant and anticancer properties. This review examined the molecular mechanisms underlying these properties and their potential application in cancer prevention and therapy. It focuses specifically on RA’s role in modulating cancer-related pathways and presents a detailed analysis of recent advancements in this area. A systematic review of PubMed, Scopus, and Web of Science databases was conducted in accordance with PRISMA (Reporting Items for Systematic Reviews and Meta-Analysis) guidelines, focusing on studies published between 2019 and 2024. A total of 25 articles providing evidence from in vitro, in vivo, and in silico studies were selected. These findings elucidate the role of RA in inhibiting tumor cell proliferation, inducing apoptosis, and preventing metastasis in various types of cancer through diverse mechanisms, including its antioxidant properties. Despite these promising results, RA’s bioavailability challenges limit its therapeutic efficacy, underscoring the necessity for improved delivery methods. This review concludes that RA exhibits significant potential as a natural agent for cancer prevention and treatment, although further clinical trials are warranted.

## 1. Introduction

Oxidative stress (OS) arises when there is an imbalance between the production of reactive oxygen species (ROS) and the ability of the body’s antioxidant defense systems to neutralize them, leading to cellular damage. ROS are chemically reactive molecules that contain oxygen, such as superoxide (O_2_^•−^), hydrogen peroxide (H_2_O_2_), and hydroxyl radicals (^•^OH), which are natural byproducts of cellular metabolism [1]. Although ROS play important physiological roles in cell signaling and defense mechanisms, their excessive accumulation can result in oxidative damage to lipids, proteins, and DNA. This damage is a critical factor in the initiation and progression of cancer, as it contributes to genetic mutations, genomic instability, and altered cellular functions [2].

At the molecular level, OS is known to induce a wide range of DNA lesions, including base modifications, strand breaks, and crosslinking [1]. One of the most prevalent types of oxidative DNA damage is the formation of 8-oxoguanine (8-oxoG), a lesion caused by ROS that leads to G- to -T transversions if left unrepaired [3]. Such mutations are frequently found in oncogenes and tumor suppressor genes and contribute to the initiation of cancer. For instance, oxidative stress-induced damage to the p53 tumor suppressor gene is commonly associated with the development of various cancers, as p53 plays a crucial role in controlling cell cycle arrest and apoptosis [4,5]. OS also plays a pivotal role in promoting cancer by activating various signaling pathways that regulate cell survival, proliferation, and differentiation. One such pathway is the nuclear factor-kappa B (NF-κB) pathway, which is sensitive to OS and is often overexpressed in cancer cells. Activation of NF-κB leads to the transcription of genes involved in inflammation, immune response, and cell survival, thereby promoting a microenvironment that favors tumor growth and resistance to apoptosis [6]. Another important pathway influenced by ROS is the mitogen-activated protein kinase (MAPK) pathway, which regulates cell proliferation and survival. Aberrant activation of this pathway by OS can promote uncontrolled cell growth and contribute to tumor progression [7].

In addition to its direct effects on cellular components, OS plays an essential role in the creation of a tumorigenic microenvironment. Chronic inflammation, which is often driven by OS, creates a favorable environment for cancer development and progression [8]. ROS can activate pro-inflammatory transcription factors, such as NF-κB, which upregulate the expression of cytokines and chemokines that sustain inflammation. This chronic inflammatory state promotes angiogenesis, tissue remodeling, and immune evasion, all of which contribute to cancer progression [8].

Moreover, OS contributes to cancer by inducing the epithelial–mesenchymal transition (EMT), a process by which cancer cells acquire invasive and metastatic properties. ROS have been shown to modulate signaling pathways involved in EMT, including the transforming growth factor-beta (TGF-β) pathway, which plays a key role in promoting tumor invasion and metastasis [9]. The enhanced migratory and invasive abilities of cancer cells driven by OS further underscore their role in the metastatic spread of cancer.

OS is a critical factor in cancer development, acting at multiple stages of tumor initiation, promotion, and progression. It plays a central role in the complex process of carcinogenesis by causing direct damage to cellular components, activating oncogenic signaling pathways and creating a tumor-promoting microenvironment [7].

Rosmarinic acid (RA) is predominantly found in a variety of medicinal and culinary herbs, especially those belonging to the Lamiaceae family, including rosemary (*Rosmarinus officinalis*), basil (*Ocimum basilicum*), sage (*Salvia officinalis*), thyme (*Thymus vulgaris*), and mints (*Mentha* spp.). In addition to the Lamiaceae family, RA is also present in plants from other families, such as Boraginaceae and Apiaceae [10]. It can also be found in various fruits and seeds, albeit in smaller quantities than herbs. Some studies have reported its presence in high-antioxidant foods, such as berries, which further extends its dietary significance as a health-promoting compound. The growing interest in natural antioxidants has led to an increased focus on RA-rich plants, not only for therapeutic purposes but also as natural preservatives in the food and cosmetic industries [11].

**Figure 1:**
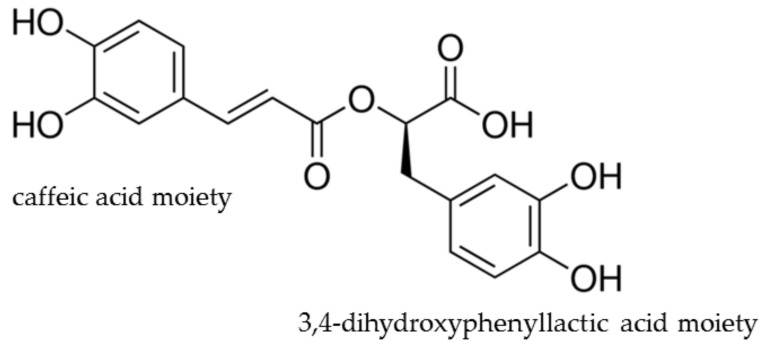
Chemical structure of RA.

RA is an ester of caffeic acid and 3,4-dihydroxyphenyllactic acid. Figure 1 shows the chemical structure of the RA; it consists of two aromatic rings with hydroxy groups that contribute to its strong antioxidant properties. Its molecular formula is C_18_H_16_O_8_, with a molecular weight of 360.32 g/mol. Its chemical structure allows it to act as a free radical scavenger by donating hydrogen atoms to stabilize ROS and free radicals. This unique antioxidant capability is largely due to the presence of a catechol moiety in its structure, which facilitates the neutralization of ROS and enhances the stability of radical intermediates [12].

RA’s dual nature as both a phenolic acid and a flavonoid-related compound enables it to chelate metal ions and prevent the formation of free radicals, thus interrupting oxidative chain reactions. Moreover, it can modulate the activity of enzymes involved in OS, such as catalase (CAT), superoxide dismutase (SOD), and glutathione peroxidase (GPx), further underscoring its potential role in preventing oxidative damage at the cellular level [13].

**Figure 2:**
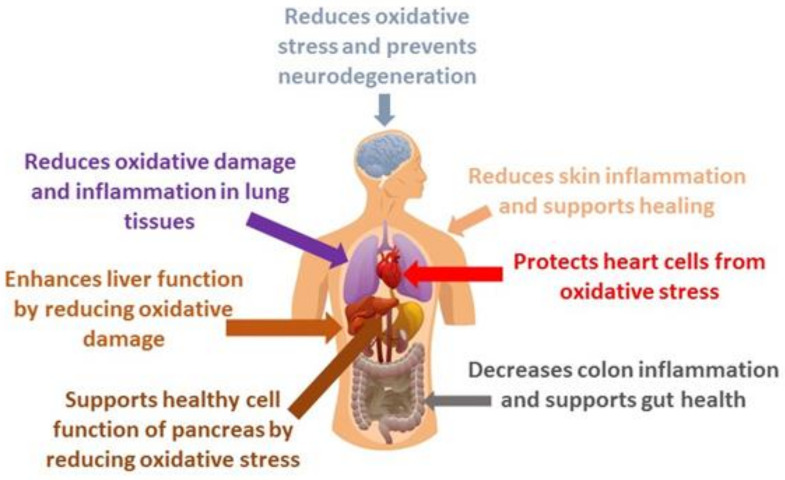
Effects of RA on the major parts of the human body.

RA is a secondary metabolite that exhibits a broad spectrum of biological activities, including antioxidant, anti-inflammatory, and antimicrobial effects, making it a compound of significant interest in both pharmacological and nutraceutical applications [12,14]. Figure 2 illustrates the broad-spectrum activities of RA on the human body.

The bioavailability of RA is a critical factor in its therapeutic efficacy, which is diminished due to its rapid metabolism and elimination from the body. Various factors, including its lipophilic nature, stability in the gastrointestinal tract, and interactions with food, can significantly influence its absorption. While RA exhibits high solubility in organic solvents, this characteristic does not ensure effective absorption in the aqueous intestinal environment. Furthermore, the stability of a compound can impact the quantity that reaches systemic circulation in its active form. To address these limitations, novel formulations for oral administration are being investigated to enhance bioavailability, including nanoparticle formulations and advanced drug delivery systems. These innovative approaches aim to protect RA from degradation and improve its absorption [12,14].

**Figure 3:**
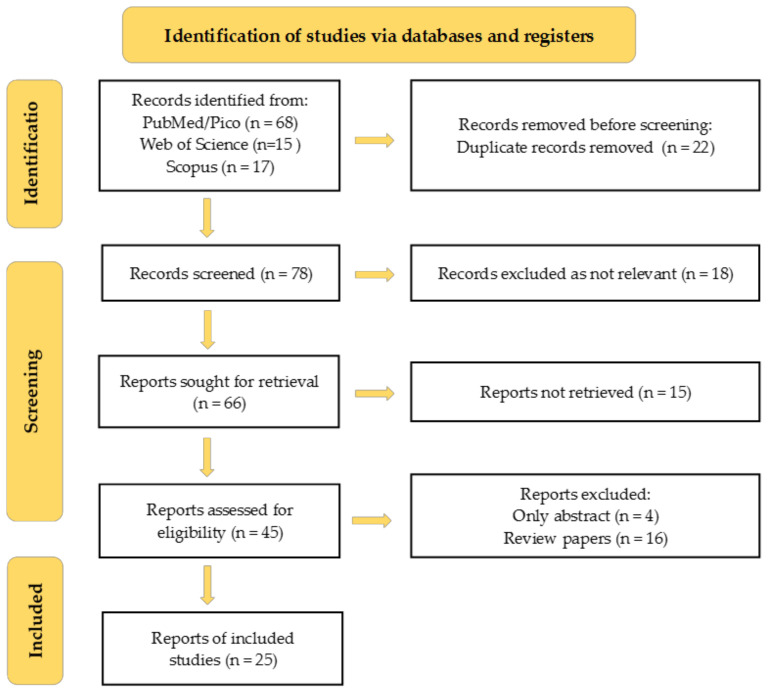
PRISMA flowchart of the included studies.

Several review articles have discussed the antioxidant properties and potential health benefits of RA in cancer. It has demonstrated significant anti-tumor potential due to its capacity to target multiple mechanisms involved in cancer progression. Studies have indicated that RA can inhibit cancer cell migration, and by inducing cell cycle arrest and promoting apoptosis, it reduces the probability of cancer growth in several organs, including the stomach, colon, liver, and breast. These actions are complemented by RA’s ability to modulate key signaling pathways responsible for cell survival and proliferation, thereby exerting a protective effect against various types of cancer. Preclinical studies have elucidated RA’s role in reducing oxidative stress and neuroinflammation, which are frequently associated with cancer progression. RA has been demonstrated to reduce lipid peroxidation and increase the activity of enzymatic antioxidants, such as superoxide dismutase and catalase, in models of oxidative stress, indicating its potential to protect against cancer-related cellular damage. RA’s capacity to modulate inflammatory responses enhances its therapeutic potential, as inflammation is a critical factor in both the development and progression of cancer [12,13,14]. This review presents a comprehensive synthesis of recent discoveries regarding the anticancer properties of RA, with a particular emphasis on its molecular mechanisms and potential therapeutic applications in cancer prevention and treatment, adhering to the PRISMA guidelines. It delineates the specific role of RA in modulating cancer-related pathways and provides a detailed analysis of recent advancements in this field. By integrating data from in vitro, in vivo, and in silico models, this study elucidates the mechanisms by which RA influences oxidative stress, inflammation, and apoptosis, thereby offering a more comprehensive understanding of its potential as an anticancer agent. To conduct this review, a systematic literature search was performed, covering studies published in English between 2019 and 2024, using databases such as PubMed/Pico, Scopus, and Web of Science, with keywords such as RA, antioxidant activity, cancer prevention, and OS. The selection process involves two stages. Initially, titles and abstracts were screened, followed by a full-text evaluation based on predefined inclusion and exclusion criteria. The inclusion criteria were studies published in English between 2019 and 2024. Studies that did not address these items, review articles, those published before 2019, and those published in languages other than English were excluded. Data extraction involved collecting information about the authors, year of publication, study objectives, model/system used, cancer type, key findings, mechanism of action and limitations. The extracted data were then synthesized and analyzed to provide a comprehensive overview. No statistical analysis was performed as this was a comprehensive literature review of scientific research. A PRISMA flowchart of the included studies is shown in Figure 3.

## 2. Mechanisms of Antioxidant Activity of RA in Cancer Diseases

The antioxidant properties of RA are derived from its capacity to scavenge free radicals, chelate metal ions and modulate antioxidant enzyme activity. Through these mechanisms, RA plays a crucial role in mitigating oxidative damage at the cellular level, thus offering protection against various diseases besides cancer, such as cardiovascular diseases, neurodegenerative disorders, and inflammatory conditions [12,14].

### 2.1. Free Radical Scavenging

Free radical scavenging is one of the primary mechanisms by which RA exerts antioxidant activity. ROS are by-products of normal cellular metabolism. Excessive ROS production leads to oxidative damage, resulting in lipid peroxidation, DNA mutations, and protein oxidation, all of which contribute to cancer and other degenerative diseases [15]. As a potent antioxidant, RA donates hydrogen atoms or electrons to neutralize free radicals, thereby preventing them from reacting with cellular components and causing damage. The chemical structure of RA, particularly the hydroxy groups on its aromatic ring, is responsible for its free radical scavenging capacity (Figure 1). These hydroxy groups function as electron donors to neutralize ROS by forming stable radicals [16]. Studies have demonstrated that RA effectively scavenges hydroxyl and superoxide radicals in vitro, significantly reducing OS in cancer cells. The study concluded that the radical scavenging capacity of RA contributes to the reduction of oxidative burden in cells exposed to external stressors, such as radiation and toxic chemicals [16,17].

### 2.2. Metal Ion Chelation

Another significant mechanism antioxidant activity of RA is its capacity to chelate transition metal ions, particularly iron (Fe^2+^) and copper (Cu^2+^), which can catalyze the formation of highly reactive hydroxyl radicals through the Fenton reaction. The chelation of metal ions inhibits their participation in these redox reactions, thereby reducing ROS generation and subsequent oxidative damage. RA has been demonstrated to effectively chelate metal ions, forming stable complexes with Fe^2+^ and Cu^2+^, thus inhibiting their pro-oxidant activity. In an in vitro study, Truong et al. [17] observed that RA effectively chelated Fe(III) ions, reducing its capacity to generate hydroxyl radicals, thus emphasizing its role as a metal ion chelator. This chelation activity is crucial because elevated levels of free iron and copper are frequently associated with increased OS and a higher risk of cancer development, particularly in tissues susceptible to metal accumulation [18]. The metal-ion binding capability of RA is primarily attributed to its catechol structure, which contains hydroxy groups capable of forming stable coordination bonds with metal ions. This property not only contributes to its antioxidant effects but also plays a role in mitigating oxidative stress-mediated damage in various pathological conditions, including neurodegenerative diseases and cancer.

### 2.3. Modulation of Antioxidant Enzymes

In addition to directly scavenging free radicals and chelating metal ions, RA enhances endogenous antioxidant defense systems by modulating the activity of antioxidant enzymes. Key antioxidant enzymes, such as SOD, CAT, and GPx, play crucial roles in neutralizing ROS and maintaining cellular redox homeostasis. RA upregulates the expression and activity of these enzymes, thereby enhancing the capacity of cells to detoxify ROS [14]. This modulation occurs through the activation of the nuclear factor erythroid 2-related factor 2 (Nrf2) pathway, a primary regulator of the antioxidant response. Nrf2 regulates the expression of various antioxidant enzymes, and the interaction of RA with this pathway results in increased expression of genes encoding SOD, CAT, and GPx. For instance, a study by Wang et al. (2021) demonstrated that RA administration in a cerebral ischemia model significantly increased the activity of SOD, CAT, and glutathione in brain tissues, alleviating OS and improving neurological outcomes [19]. Similarly, Lu et al. (2022) observed that RA supplementation enhanced the antioxidant defense by activating the Nrf2 pathway in a carbon tetrachloride-induced liver injury model, increasing the levels of SOD and CAT, and reducing OS markers [20]. This ability to modulate antioxidant enzymes makes RA a potent agent for protecting cells from oxidative damage, particularly in conditions such as cancer, where OS plays a critical role in disease progression.

### 2.4. RA and Oxidative Stress: How RA Mitigates Oxidative Damage at the Cellular Level

At the cellular level, RA has been demonstrated to mitigate oxidative damage to DNA by neutralizing ROS prior to their interaction with DNA molecules. Li et al. (2019) reported that RA treatment significantly reduced the levels of 8-oxoguanine, a common marker of oxidative DNA damage, in cancer cells exposed to OS [21]. The reduction in DNA damage not only protects cells from mutations that can lead to cancer but also enhances the cell’s repair mechanisms, allowing damaged DNA to be corrected before it becomes a permanent mutation. Furthermore, the interaction between RA and the Nrf2/Keap1 pathway plays a crucial role in maintaining cellular redox homeostasis. By activating Nrf2, RA enhances the expression of several phase II detoxifying enzymes that are essential for neutralizing ROS and reducing oxidative damage [22]. Activation of the Nrf2 pathway has been associated with improved cell survival and reduced cancer risk in models of oxidative stress-induced carcinogenesis.

RA also protects cellular lipids from peroxidation, which can disrupt membrane integrity and lead to cell death. Studies have demonstrated that RA significantly reduces lipid peroxidation in cells exposed to OS, thereby preserving membrane stability and function [21]. This protection is vital for maintaining cell viability under OS, particularly in cancer cells, where oxidative damage to membranes can trigger apoptosis.

The ability of RA to mitigate OS at the cellular level results from its multifaceted antioxidant activity. By scavenging ROS, chelating metal ions, modulating antioxidant enzyme activity, and activating protective pathways, such as Nrf2, RA reduces oxidative damage to DNA, proteins, and lipids, thereby protecting cells from the harmful effects of OS. This protection is especially important in cancer prevention and therapy, in which OS plays a significant role in the development and progression of the disease.

## 3. Experimental Evidence

As summarized in Table 1, preclinical studies have investigated the effects of RA on diverse cancer cell lines, animal models, or in silico. These investigations demonstrated that RA is a multifaceted agent capable of inducing apoptosis, inhibiting metastasis, and modulating OS.

### 3.1. In Vitro Studies

In vitro research on RA has been crucial in understanding its potential as an anticancer agent across various cancer cell lines. The studies summarized in Table 1 provide insights into RA’s mechanisms of action, particularly in its ability to modulate cancer cell proliferation, induce apoptosis, and inhibit metastasis. However, these findings require further exploration in vivo models and clinical studies.

#### 3.1.1. Skin Cancer

The study conducted by Rodríguez-Luna [23] investigated the effects of RA in combination with fucoxanthin in human HaCaT keratinocytes, a model for UV radiation-induced skin cancer. The results demonstrated that RA and fucoxanthin reduced UVB-induced apoptosis and inflammation by downregulating inflammasome components, such as NLRP3, ASC, and Caspase-1, while upregulating Nrf2 and HO-1. These findings suggest a potential protective role of RA in skin cancer prevention, particularly in mitigating the harmful effects of UV radiation. In 2016, Hasegawa et al. explored the UVB-induced inflammatory responses and NLRP3 inflammasome activation in human keratinocytes [48]. Both studies found that UVB exposure increased NLRP3 activation and reported the involvement of pro-inflammatory markers, such as IL-1β, which are regulated by the NLRP3 inflammasome. Rodríguez-Luna et al. demonstrated a reduction in IL-1β production through the downregulation of NLRP3, suggesting a protective and therapeutic role for RA and fucoxanthin in skin cancer prevention. Gupta et al. [24] investigated the role of RA in preventing UVB-induced skin cancer by studying its effects on human skin cells, specifically primary human dermal fibroblasts and HaCaT keratinocytes. This study demonstrated that RA reduced UVB-induced mitochondrial fragmentation and oxidative imbalance, which are key contributors to the development of skin cancer. RA appears to protect skin cells from the damaging effects of UV radiation by modulating mitochondrial dynamics and ROS levels, suggesting a potential preventive role of RA in skin cancer caused by sun exposure. A study conducted by Fernando et al. [49] examined the cytoprotective effects of RA against UVB-induced OS in HaCaT keratinocytes. The investigation revealed that RA significantly reduced UVB-induced intracellular ROS and mitigated oxidative damage to proteins, DNA, and lipids. Furthermore, RA enhanced the activity of antioxidant enzymes, such as SOD, CAT, and heme oxygenase-1 (HO-1), by activating the Nrf2 pathway, a critical regulator of the cellular antioxidant response. These findings align with Gupta’s results, highlighting the role of RA in modulating OS and enhancing cellular defense mechanisms against UVB radiation

#### 3.1.2. Breast Cancer

Sevimli-Gur et al. [25] examined the effects of RA in multiple cancer cell lines, including breast, colon, cervical, prostate, and neuroblastoma cells. Notably, RA promoted cell proliferation in most cell lines, particularly in breast cancer cells (MCF-7), where it exhibited the highest IC50. This observation raises concerns regarding the potential of RA to act as a growth factor in certain cancers, contradicting previous studies that emphasize its cytotoxic properties. These contradictory results suggest that the effects of RA may be highly context-dependent and vary significantly depending on cancer type, cell line, and experimental conditions. Breast cancer studies focused on RA also include the work of Anwar et al. [26] and Ghiulai et al. [27]. Anwar’s study demonstrated that RA inhibited MARK4 activity in MDA-MB-231 breast cancer cells, resulting in dose-dependent apoptosis [26]. MARK4, a kinase involved in cancer progression, was effectively targeted by RA, suggesting its potential as a promising therapeutic target for breast cancer. Ghiulai’s study similarly observed that RA exhibited dose-dependent inhibition of breast cancer cell growth, particularly in estrogen-dependent MCF7 cells, and induced both apoptosis and autophagy [27]. These dual mechanisms of cell death underscore the potential versatility of RA as an anticancer agent, particularly for hormone-responsive cancers. Khanaree et al. [28] investigated the effects of RA on metastasis in MDA-MB-231 breast cancer cells, focusing on its anti-metastatic properties. The study demonstrated that RA effectively inhibited cancer cell invasion and migration by reducing matrix metalloproteinase-9 (MMP-9) activity. MMP-9 is a proteolytic enzyme crucial for the breakdown of the extracellular matrix, which facilitates cancer metastasis. By inhibiting this enzyme, RA may help prevent the spread of cancer, particularly in advanced-stage cancers. Cristy et al. [29] investigated a novel delivery method for RA utilizing RA-loaded microemulsions in breast cancer cells (T47D and MDA-MB-231). This investigation revealed that these microemulsions demonstrated superior antioxidant activity compared to free RA, inhibited cell proliferation, induced apoptosis, and caused cell cycle arrest. This enhanced therapeutic effect was attributed to the improved bioavailability and stability of RA when delivered via microemulsions, potentially overcoming the limitations associated with RA’s poor solubility and rapid degradation in biological systems. Previous research conducted by Li et al. [50] investigated the effects of RA from *Sarcandra glabra* on MDA-MB-231 breast cancer cells. This study found that RA inhibited cell proliferation and migration in a dose- and time-dependent manner while also inducing apoptosis. Mechanistically, RA downregulates Bcl-2 expression and upregulates Bax, thereby promoting apoptosis. This further supports the hypothesis that the apoptotic effects of RA in breast cancer cells are mediated through the regulation of key apoptotic proteins, such as Bcl-2 and Bax. Like Anwar’s and Ghiulai’s research, Li’s study emphasizes the importance of apoptosis induction in RA’s anticancer properties but extends it by providing insights into the specific molecular pathways involved. His study found that MMP-9 was predominantly expressed in the areas of active cancer cell proliferation and metastasis, whereas MMP-2 was linked to apoptosis in different bone zones. This aligns with Khanaree’s findings on RA, reinforcing the notion that MMP-9 plays a critical role in breast cancer metastasis and that its inhibition could be a vital therapeutic target. Li et al.’s work corroborates Cristy’s conclusions by demonstrating that RA is effective in inhibiting cancer progression, further validating the potential of RA-loaded microemulsions in enhancing RA’s therapeutic effects through improved delivery mechanisms.

#### 3.1.3. Pancreatic and Liver Cancer

Han et al. [30] reported that RA suppressed cell viability, migration, and invasion in pancreatic cancer cell lines (Panc-1 and SW1990) while promoting apoptosis. The study identified the upregulation of miR-506 and the suppression of MMP2 and MMP16 as the mechanisms by which RA inhibits epithelial–mesenchymal transition (EMT), a critical process in cancer metastasis. These findings are consistent with those reported by Wang [31], who demonstrated that RA inhibited the growth, invasion, and metastasis of hepatocellular carcinoma (HCC) cells by regulating matrix metalloproteinases (MMP-2 and MMP-9) and promoting E-cadherin expression, while downregulating N-cadherin and vimentin. Both studies suggested that RA’s capacity to target EMT-related mechanisms could be efficacious in treating metastatic cancers. Zhou et al. [32] investigated the effects of RA on pancreatic ductal adenocarcinoma (PDAC), one of the most aggressive and treatment-resistant cancers. Zhou’s study demonstrated that RA inhibited PDAC cell proliferation and induced apoptosis by downregulating Gli1, a key component of the Hedgehog signaling pathway, which plays a crucial role in cancer cell survival and proliferation. Furthermore, RA induced G1/S cell cycle arrest, preventing cancer cells from progressing through the cell cycle, which is essential for cell growth and division. These findings highlight the potential of RA to target the signaling pathways critical for the survival of aggressive cancers, such as pancreatic cancer.

#### 3.1.4. Colorectal Cancer

Laila et al. [33] investigated colorectal cancer using the WiDr colon cancer cell line and observed that RA demonstrated antiproliferative effects and induced apoptosis. This effect was attributed to the downregulation of BCL2 and the upregulation of Caspase 1 and Caspase 7, which are critical for apoptotic pathways. Similarly, Yang et al. [34] demonstrated that RA inhibits the migration and invasion of HT-29 colorectal cancer cells by modulating miR-1225-5p and regulating the Nrf2/Keap1 pathway. This pathway plays a significant role in cellular defense against OS, and RA’s ability to attenuate p38/AP-1 signaling via IL-17RA targeting provides further evidence of its anticancer potential. A previous study conducted by Jang et al. [51] explored the impact of RA on prostate cancer cell lines and found that RA induced apoptosis by modulating histone deacetylase 2 (HDAC2) expression. This process involves the downregulation of BCL2 and upregulation of apoptotic markers, such as Bax and caspase-3. The mitochondrial pathway of apoptosis has been highlighted as a key mechanism through which RA exerts its anticancer effects. These findings align with the conclusions of Laila and Yang on RA’s role in regulating BCL2 and caspases, further demonstrating RA’s ability to induce apoptosis in different cancer type. Also, Yang et al. [34] and Jin et al. [35] both investigated the effects of RA on colorectal cancer, with Jin focusing on colitis-associated colorectal cancer. Jin’s study demonstrated that RA reduced tumor incidence and inflammation by suppressing TLR4-mediated NF-κB-STAT3 signaling, which plays a crucial role in inflammation and tumorigenesis in the colon. Yang’s study provided complementary findings, indicating that RA inhibited migration and invasion in colorectal cancer cells by regulating miR-1225-5p and modulating the Nrf2/Keap1 pathway. A study conducted by Xu et al. [52] examined the anti-Warburg effect of RA in colorectal carcinoma, focusing on its ability to suppress glucose consumption and lactate generation in colorectal carcinoma cells. This study demonstrated that RA inhibits hypoxia-inducible factor-1 alpha (HIF-1α) and downregulates miR-155, which affects the IL-6/STAT3 pathway, thereby reducing inflammation and promoting apoptosis in cancer cells. Xu’s findings align with those of Jin’s study, further validating the role of RA in modulating the NF-κB-STAT3 signaling pathway, which is crucial for inflammation-driven tumorigenesis.

#### 3.1.5. Oral Cancer

Luo et al. [36] demonstrated that RA inhibits proliferation, induces apoptosis, and reduces migration in SCC-15 oral cancer cells. These effects were mediated by the induction of ER stress and inhibition of MMP-2 and MMP-9, further supporting the role of RA in targeting epithelial–mesenchymal transition (EMT). Previous research conducted by Radziejewska et al. [53] investigated the effects of RA on gastric adenocarcinoma cells and found that RA also inhibited MMP-9 activity. The study examined how RA modulates MMPs, TIMPs (tissue inhibitors of metalloproteinases), and glycosylation in cancer cells. This regulation of extracellular matrix components by RA is significant because MMPs, such as MMP-2 and MMP-9, play essential roles in cancer metastasis by degrading collagen and other proteins in the extracellular matrix. The ability of RA to reduce MMP-9 activity is consistent with Luo et al.’s findings, highlighting its potential to inhibit cancer cell migration and invasion.

#### 3.1.6. Osteosarcoma

Ma et al. [37] investigated the effects of RA on osteosarcoma cell lines (U2OS and MG63) and demonstrated that RA inhibited proliferation, migration, and invasion while inducing apoptosis through the generation of ROS. This study identified the PTEN-PI3K-Akt signaling pathway as a key mechanism by which RA regulates OS and inhibits cancer progression. These findings elucidate the potential of RA as a treatment for osteosarcoma, particularly through its ability to modulate the OS pathways. A previous study conducted by Lin et al. [54] examined RA’s capacity to protect rat bone marrow mesenchymal stem cells from hydrogen peroxide-induced apoptosis. This study found that RA reduced ROS production, protected cells from apoptosis-related morphological changes, and regulated apoptosis-related proteins, such as caspase-3, caspase-9, Bax, and Bcl-2. Notably, RA has been shown to upregulate p-PI3K, protecting cells through the PI3K/Akt pathway, a mechanism also discussed in Ma’s study. This research further supports the role of RA in modulating OS and inhibiting apoptosis in various cell types, reinforcing its potential as an anti-cancer agent.

#### 3.1.7. Lung Cancer

Lung cancer research by Pintha et al. [38] and Tantipaiboonwong et al. [39], who investigated the extract from *Perilla frutescens* effects RA in A549 lung adenocarcinoma cells, has shown promising findings. Pintha et al. observed that RA-rich fractions reduced OS, inflammation, and metastasis in lung cancer cells exposed to particulate matter, implicating pathways such as C-Jun, P-65-NF-κB, and Akt. Similarly, Tantipaiboonwong et al. demonstrated that RA reduced TNF-α-induced OS and inflammation, suggesting that RA could attenuate inflammatory responses in lung cancer. A previous study by Moon et al. [55] demonstrated that RA sensitized TNF-α-induced apoptosis in human leukemia U937 cells through the suppression of NF-κB activation and ROS generation. RA inhibition of NF-κB, along with its ability to lower ROS levels, leads to increased activation of caspase-dependent apoptosis, which consequently reduces cancer cell survival. Although Moon’s study focused on leukemia cells, it aligns with the results from Pintha and Tantipaiboonwong by elucidating RA’s potential to inhibit key pathways involved in inflammation and OS, mechanisms that are also central to lung cancer progression.

Punfa et al. [40] investigated the effects of RA on non-small cell lung carcinoma (NSCLC) in the A549 cell line. This study demonstrated that RA reduced ROS production and pro-inflammatory mediator expression, both of which are implicated in cancer progression and metastasis. By reducing ROS, RA appears to exert a protective effect against OS, which frequently contributes to tumor growth and metastasis. A previous study by Liu et al. [56] have provided further evidence supporting the role of ROS in mediating apoptosis in A549 lung adenocarcinoma cells. Their research revealed that *Polygonatum cyrtonema* lectin (PCL), similar to RA, induces significant ROS generation in A549 cells, which triggers both apoptosis and autophagy. Moreover, the study demonstrated that inhibiting ROS production with scavengers reduces apoptosis, suggesting that ROS plays a central role in mediating cancer cell death in response to therapeutic agents such as RA. Liu et al. also found that the MAPK and NF-κB pathways are critical in regulating ROS-induced apoptosis, further linking OS with key cancer survival pathways. RA enhanced the activity of antioxidant enzymes like SOD and CAT, while reducing lipid peroxidation. This mitigated OS, a factor known to contribute to cancer development. Additionally, RA reduced the expression of inflammatory mediators, further supporting its anticancer properties [41].

#### 3.1.8. Cardioprotection in Chemotherapy

Rahbardar et al. [42] investigated the protective effects of RA on doxorubicin-induced cardiotoxicity in rats, related to breast cancer treatment on MCF7 cell lines, focusing on mitigating OS and enhancing hemodynamic parameters. The study emphasized that while doxorubicin is an efficacious chemotherapeutic agent, its utilization is frequently constrained by its cardiotoxic side effects, which can induce substantial cardiac damage. RA attenuated these effects by reducing ROS levels, indicating its potential role as a cardioprotective agent during chemotherapy. Similarly, Sunitha et al. [57] demonstrated the cardioprotective properties of plant-derived compounds, showing that RA-like compounds can mitigate doxorubicin-induced toxicity through mechanisms involving OS reduction. These studies highlight RA’s potential in reducing the harmful side effects of chemotherapy on the heart.

#### 3.1.9. Prostate Cancer

Garcia et al. [44] investigated the anticancer potential of RA in prostate cancer, specifically utilizing DU-145 prostate cancer cell lines. This study demonstrated that RA-rich extracts from *Perilla frutescens* significantly inhibited cell proliferation, migration, and invasion, while inducing apoptosis. These findings are noteworthy, particularly considering the role of metastasis in cancer-related mortality. By inhibiting cancer cell migration and invasion, RA exhibits potential to impede the spread of cancer to other organs and improve patient outcomes in advanced prostate cancer. A previous study conducted by Jang et al. [51] examined the effects of RA on the prostate cancer cell lines PC-3 and DU-145. The study found that RA induced cell cycle arrest and apoptosis by modulating the expression of HDAC2, a histone deacetylase that is typically overexpressed in prostate cancer and is associated with tumor progression. RA downregulation of HDAC2 results in the activation of p53, a tumor suppressor protein, leading to increased apoptosis in prostate cancer cells. These findings align with Garcia’s study, reinforcing the hypothesis that RA’s capacity to inhibit cell proliferation and induce apoptosis could be crucial in treating aggressive prostate cancer.

### 3.2. In Vivo Studies

While in vitro studies provide crucial insights into the cellular mechanisms of RA, in vivo studies are essential to elucidate its functions in complex biological systems. In vivo research in 2019–2024 has demonstrated its potential as an anticancer agent across multiple cancer types. Various studies employed animal models to assess RA’s efficacy in inhibiting tumor growth, reducing inflammation, and improving survival rates. These models included xenograft mouse models, BALB/c nude mice, and rat models

For pancreatic cancer, both Han et al. [30] and Zhou et al. [32] using the xenograft mouse model, reported significant tumor suppression with RA treatment. Han’s study demonstrated that RA inhibited tumor growth in a dose-dependent manner by regulating the miR-506/MMP2/16 axis, while Zhou’s research focused on RA’s impact on inhibiting Gli1 signaling, which plays a crucial role in pancreatic ductal adenocarcinoma progression. In hepatocellular carcinoma (HCC) on male BALB/c nude mice, Wang [31] found that RA reduced tumor volume and increased apoptosis rates by inhibiting the PI3K/AKT/mTOR signaling pathway, which is essential for cancer cell survival and proliferation [31]. Moreover, on the Ehrlich solid carcinoma mouse model in breast cancer, Mahmoud et al. [43] found that RA enhanced the therapeutic effects of paclitaxel, primarily through modulation of the NF-κB, TNF-α, VEGF, and p53 pathways. For colorectal cancer, both Jin [35] on the AOM/DSS-induced CAC mouse model and Ilhan et al. [45] on a rat model confirmed that RA significantly inhibited tumor formation. Jin’s study, which focused on colitis-associated colorectal cancer (CAC), found that RA reduced tumor incidence by modulating TLR4-mediated NF-κB and STAT3 signaling pathways. These results are in line with Cao et al. [58], who also demonstrated RA’s anti-inflammatory and anti-tumor effects through NF-κB and Bcl-2 regulation in hepatocellular carcinoma. Glioblastoma multiforme studies on a rat model, such as Khaksar et al. [46], further support RA’s broad anticancer activity. Khaksar’s research showed that RA reduced tumor volume and improved survival rates by inducing apoptosis and suppressing NF-κB signaling. This is consistent with findings from other studies on breast and colorectal cancer, where RA’s inhibition of NF-κB was identified as a key mechanism in suppressing tumor growth [43]. Such results suggest that RA’s effects on the NF-κB pathway may be a common mechanism across various cancers, making it a versatile candidate for further therapeutic development.

### 3.3. In Silico Studies

In silico research has become an indispensable tool in drug discovery, particularly in evaluating compounds such as RA for their potential in cancer treatment. Computational models, including molecular docking and molecular dynamics simulations, provide early insights into drug efficacy and molecular mechanisms of action. These methods are invaluable in predicting RA’s interactions with key proteins involved in cancer progression, offering a cost-effective and time-efficient alternative to traditional experimental studies.

Anwar et al. [26] demonstrated that RA binds with high affinity to the MARK4 (microtubule affinity-regulating kinase 4) protein, which is strongly associated with breast cancer. Their molecular docking and 500 ns all-atom simulations revealed that RA forms stable non-covalent interactions with critical residues in the MARK4 active site, suggesting that RA may inhibit MARK4’s role in cancer progression. This is supported by experimental evidence that RA can inhibit cancer cell growth and induce apoptosis in MARK4-related tumors. These results align with findings from Highland et al. [59], who showed that RA also targets the focal adhesion kinase (FAK) in non-small cell lung cancer (NSCLC). Their study highlighted RA’s ability to form stable complexes with FAK, thereby inhibiting metastasis-associated signaling pathways. This demonstrates RA’s potential not only in inhibiting MARK4 in breast cancer but also in arresting metastasis in other cancers through similar protein-interaction mechanisms.

Li et al. [47] expanded the scope of RA’s anticancer potential by focusing on liver cancer. Using molecular docking and dynamics simulations, Li’s study investigated RA’s interaction with HSP90AA1, a protein critical for cancer cell survival and proliferation in liver cancer. The research revealed that RA binds with high affinity to HSP90AA1 and forms stable hydrogen bonds at the active site. This suggests that RA may inhibit the oncogenic activities of HSP90AA1, thereby impairing cancer cell survival. The inhibition of HSP90AA1 disrupts multiple cancer-related pathways, further emphasizing RA’s potential as a therapeutic agent. These findings are consistent with those of Cao et al. [58], who explored the anti-inflammatory and anti-angiogenic effects of RA in hepatocellular carcinoma (HCC). Their study showed that RA suppresses inflammation-related cytokines and angiogenesis by downregulating NF-κB signaling in liver tumors, suggesting a similar mode of action to its inhibition of HSP90AA1 in liver cancer. This parallel between the inhibition of different cancer pathways highlights RA’s versatility as an anticancer compound.

## 4. Challenges and Future Directions

Despite the promising preclinical results of RA, several challenges persist in the translation of these findings into clinical practice. A significant limitation of RA is its poor bioavailability. Following oral administration, RA undergoes rapid metabolism and excretion, resulting in low systemic concentrations, which restrict its therapeutic efficacy [60]. To address this issue, novel delivery systems, such as nanoparticle encapsulation and liposomal formulations, should be developed to enhance the stability and absorption of RA in cancer therapy [11,29,61,62]. These systems aim to improve the bioavailability of RA and facilitate the achievement of therapeutic concentrations in target tissues. Moreover, while RA has shown promising anti-tumor activity in monotherapy, there is growing interest in its use in combination with conventional therapies. For example, RA’s ability to alleviate OS and inflammation makes it a promising candidate for combination with chemotherapy or radiotherapy, potentially increasing treatment efficacy while attenuating side effects. Future clinical trials should focus on optimizing RA combination therapies and testing their long-term safety and efficacy in humans. Finally, there are still gaps in our understanding of the molecular mechanisms underlying RA. Although RA has been shown to modulate multiple signaling pathways involved in cancer progression, further research is needed to elucidate specific molecular targets in different cancer types. This knowledge could help tailor the use of RA to specific cancer subtypes and enhance its therapeutic efficacy.

## 5. Conclusions

In the presented studies, RA has shown substantial potential as an anticancer agent in numerous preclinical studies, with evidence supporting its ability to induce apoptosis, inhibit proliferation, and modulate inflammation and metastasis across various cancer types. RA’s anticancer properties have been demonstrated in in vitro, in vivo, and in silico models, particularly through the activation of apoptotic pathways and the inhibition of key enzymes, like MMP-2 and MMP-9, which are involved in cancer metastasis. One of the most promising aspects of RA is its ability to regulate both OS and inflammation, critical factors in cancer development, especially in inflammation-related cancers, such as colorectal cancer. By downregulating pro-inflammatory pathways, like NF-κB and STAT3, RA demonstrates potential in preventing cancer progression while mitigating oxidative damage through its antioxidant effects. Despite these promising findings, the clinical application of RA is limited by its poor bioavailability. RA undergoes rapid metabolism, which reduces its effectiveness in systemic circulation. To address this challenge, innovative drug delivery systems, including nanoformulations and other carriers, are being developed to enhance RA’s stability and absorption. Moreover, RA holds potential as a complementary therapy in combination with conventional cancer treatments, like chemotherapy, where it could reduce side effects and enhance therapeutic efficacy. Future research should focus on overcoming bioavailability issues, advancing clinical trials, and exploring RA’s molecular mechanisms further. With these advancements, RA could emerge as a valuable tool in cancer prevention and treatment. While RA’s preclinical results are promising, its full potential in cancer therapy can only be realized through continued research and clinical validation.

## Figures and Tables

**Table 1:** Summary of reviewed articles on the anticancer effects of RA.

Ref.	Source of RA	Study Type	Model/System Used	Cancer Type	Key Findings	Mechanism of Action	Limitations
[23]	Commercial substance	In vitro	Human HaCaT keratinocytes	Skin cancer (UV radiation-related)	RA + fucoxanthin reduced UVB-induced apoptosis and inflammation	Downregulation of inflammasome components (NLRP3, ASC, Caspase-1) and IL-1β; upregulation of Nrf2 and HO-1	Did not explore long-term effects or efficacy in vivo
[24]	Commercial substance	In vitro	Human skin cells (Primary Human Dermal Fibroblasts, HaCaT keratinocytes)	Skin cancer (UVB exposure-related)	RA reduced UVB-induced mitochondrial fragmentation and oxidative imbalance; reduced apoptosis	Modulation of mitochondrial dynamics; reduction of ROS levels	Limited to in vitro models
[25]	Commercial substance	In vitro	Multiple human cancer cell lines	Breast, colon, cervix, prostate, neuroblastoma	RA promoted proliferation in most cell lines tested; highest IC50 in MCF-7 cells	Complex interaction with cancer cells; RA may promote cell proliferation rather than cytotoxicity	Limited to in vitro; no long-term studies or interaction with other compounds
[26]	Commercial substance	In vitro; In silico	MDA-MB-231 breast cancer cell line; Molecular docking and all-atom simulation	Breast cancer	RA inhibits MARK4 activity, induces dose-dependent apoptosis, high binding affinity to MARK4	RA inhibits MARK4 activity and binds to the active site of MARK4, forming non-covalent interactions with critical residues	Results may not fully represent in vivo conditions; potential effects on other cell types not assessed; computational models require validation
[27]	*Melissa officinalis* extract	In vitro; In vivo	MCF7, MDA-MB-231 breast cancer cell lines; Chorioallantoic membrane assay	Breast cancer	RA exhibited dose-dependent inhibition of breast cancer cell growth, especially in estrogen-dependent cells	Induction of apoptosis and autophagy; suppression of inflammatory responses involving proapoptotic and antiangiogenic effects	Cytotoxic effects and interaction with other compounds need clarification; tumor microenvironment complexity
[28]	*Perilla frutescens* extract	In vitro	MDA-MB-231 human breast cancer cells	Breast cancer	RA exhibited anti-metastatic properties by inhibiting invasion and migration; reducing MMP-9 activity	Inhibition of proteolytic enzymes; reduction of basement membrane breakdown	Focused on in vitro; needs in vivo and clinical research
[29]	Commercial substance	In vitro	Human breast cancer cells (T47D, MDA-MB-231)	Breast cancer	RA-loaded microemulsions exhibited superior antioxidant activity; inhibited proliferation	Induced apoptosis; cell cycle arrest; enhanced antioxidant activity	Further studies needed on microemulsion carriers; limited to in vitro
[30]	Commercial substance	In vitro; In vivo	Panc-1, SW1990 pancreatic cell lines; xenograft mice	Pancreatic cancer	RA suppressed cell viability, migration, and invasion; promoted apoptosis; dose-dependently suppressed tumor growth	Up-regulation of miR-506 and suppression of MMP2 and MMP16 expression, leading to inhibition of epithelial-mesenchymal transition (EMT)	Needs further exploration of additional targets of miR-506 and applicability to human biology; clinical studies needed to validate findings
[31]	Commercial substance	In vitro; In vivo	Human hepatocellular carcinoma cell line SMMC-7721; Male BALB/c nude mice	Hepatocellular carcinoma (HCC)	RA significantly inhibited proliferation, induced G1 arrest, and promoted apoptosis; reduced tumor volume in mice	Inhibition of PI3K/AKT/mTOR signaling pathway affecting cell proliferation and survival	Potential differences between in vitro and in vivo responses; animal models may not represent human physiology; safety in humans not established
[32]	Commercial substance	In vitro; In vivo	Human PDAC cell lines (PATU-8988, MIA PaCa-2, PANC-1, BxPC-3); xenograft mouse model	Pancreatic ductal adenocarcinoma (PDAC)	RA significantly decreased cell viability; induced apoptosis and inhibited cell proliferation in PDAC cell lines; suppressed tumor growth in mice	Downregulation of Gli1; induction of G1/S cell cycle arrest; regulation of apoptosis-related proteins	Molecular mechanisms need further exploration for clinical applicability; further research needed to confirm long-term effects and safety
[33]	*Coleus amboinicus* extract	In vitro	WiDr colon cancer cell line	Colorectal cancer	RA exhibited antiproliferative effects, induced apoptosis	Downregulation of BCL2; upregulation of Caspase 1 and Caspase 7	Needs validation in animal models or clinical settings
[34]	Commercial substance	In vitro	HT-29 human colorectal cancer cell line	Colorectal cancer	RA inhibited migration and invasion of colorectal cancer cells	Regulation of miR-1225-5p; modulation of Nrf2/Keap1; attenuation of p38/AP-1 signaling via IL-17RA targeting	Primarily in vitro; needs validation in vivo and clinical settings
[35]	Commercial substance	In vitro; In vivo	Human colorectal carcinoma cell lines (HCT116, HT29); AOM/DSS-induced CAC mouse model	Colorectal cancer (colitis-associated)	Inhibited cell proliferation, reduced inflammatory cytokine production; reduced tumor incidence in mouse model	Suppression of TLR4-mediated NF-κB-STAT3 signaling	Limited generalizability to human conditions; clinical trials needed
[36]	Commercial substance	In vitro	Normal hTRET-OME oral cell line; SCC-15 oral cancer cell line	Oral cancer	RA inhibited proliferation, induced apoptosis, caused G2/M arrest, reduced migration	Induction of ER stress; inhibition of MMP-2 and MMP-9	Limited to in vitro studies; in vivo validation required
[37]	Commercial substance	In vitro	Human osteosarcoma cell lines (U2OS, MG63)	Osteosarcoma	RA inhibited proliferation, migration, and invasion; induced apoptosis through ROS production	Regulation of OS; inhibition of DJ-1 via PTEN-PI3K-Akt signaling pathway	Limited to in vitro models
[38]	*Perilla frutescens* extract	In vitro	Human lung epithelial A549 cells	Lung cancer	RA-rich fraction reduced OS, inflammation, and metastasis in lung cancer cells exposed to particulate matter	Involvement of C-Jun, P-65-NF-κB, and Akt signaling pathways	Limited to in vitro; in vivo validation required
[39]	*Perilla frutescens* extract	In vitro	A549 lung adenocarcinoma cells	Lung adenocarcinoma	RA reduced TNF-α-induced OS and inflammation	Inhibition of pro-inflammatory cytokine production; modulation of OS pathways	Limited to in vitro; requires animal model validation
[40]	*Perilla frutescens* extract	In vitro	A549 human lung carcinoma cells	Non-small cell lung carcinoma	RA reduced ROS production and pro-inflammatory mediator expression	Reduction of ROS; inhibition of inflammatory pathways	Limited to in vitro; further research needed in animal models and clinical settings
[41]	Commercial substance	In vivo	Mouse model (radiation-induced pulmonary fibrosis)	Thoracic cancer (related to radiation therapy)	RA exhibited antifibrotic effects; inhibited fibroblast-to-myofibroblast transition	Modulation of SPHK1 and PFKFB3 acetylation; reduction of glycolysis-triggered fibroblast transition	Needs further human clinical trials to validate findings
[42]	Commercial substance	In vitro; In vivo	MCF7 breast cancer cell lines; Doxorubicin-induced cardiotoxicity in rats	Breast cancer	RA ameliorated doxorubicin-induced cardiotoxicity; improved heart weight/body weight ratio and hemodynamic parameters	Antioxidant properties; modulation of OS and cardiac tissue histology; reduction of OS and improvement of hemodynamic factors	High concentrations of RA may induce cytotoxic effects; further research needed to elucidate detailed mechanisms, bioavailability, and optimal dosing
[43]	Commercial substance	In vivo	Ehrlich solid carcinoma mouse model	Breast cancer	RA showed chemo-preventive and therapeutic potential; enhanced effects of Paclitaxel	Modulation of NF-κB, TNF-α, VEGF; regulation of p53, Bcl-2, Bax, Caspase-3	Focused on single animal model; results may not generalize to other cancer types
[44]	*Perilla frutescens* extract	In vitro	Prostate cancer cell lines (DU-145)	Prostate cancer	RA-rich extract showed significant cytotoxicity; inhibited proliferation, migration, and invasion; induced apoptosis	Inhibition of cancer cell proliferation, migration, and invasion; induction of apoptosis	Primarily in vitro; needs validation in vivo and clinical settings
[45]	Commercial substance	In vivo	Rat model (AOM-induced colorectal cancer)	Colorectal cancer (CRC)	RA significantly inhibited tumor formation; improved oxidant-antioxidant status	Antioxidant and anti-inflammatory properties	Molecular mechanism needs further elucidation; requires advanced studies
[46]	Commercial substance	In vivo	Rat model (glioblastoma multiforme)	Glioblastoma multiforme	RA reduced tumor volume; improved survival rates; influenced OS markers and apoptosis pathways	Induction of apoptosis; inhibition of cell proliferation; suppression of NF-κB inflammatory signaling	Conducted in animal model; human research needed to explore long-term effects
[47]	*Cordia myxa* extract	In silico	Molecular docking dynamics simulations	Liver cancer	RA showed higher binding affinity to HSP90AA1 protein compared to control drug	Formation of stable hydrogen bonds with HSP90AA1 protein; inhibition of cancer-related signaling pathways	Relies on computational models; experimental validation needed
